# Chronic stress-induced depression requires the recruitment of peripheral Th17 cells into the brain

**DOI:** 10.1186/s12974-022-02543-6

**Published:** 2022-07-14

**Authors:** Zhuang Peng, Sha Peng, Kangguang Lin, Bin Zhao, Lai Wei, Qinhui Tuo, Duanfang Liao, Tifei Yuan, Zhe Shi

**Affiliations:** 1 Key Laboratory for Quality Evaluation of Bulk Herbs of Hunan Province, Hunan University of Chinese Medicine, Changsha, Hunan China; 2 Department of Affective Disorders, The Affiliated Brain Hospital of Guangzhou Medical University (Guangzhou Huiai Hospital), Guangzhou, Guangdong China; 3 Xinxiang Key Laboratory of Forensic Toxicology, School of Forensic Medicine, Xinxiang Medical University, Xinxiang, Henan China; 4 Shanghai Key Laboratory of Psychotic Disorders, Shanghai Mental Health Center, Shanghai Jiao Tong University School of Medicine, Shanghai, China; 5 Co-Innovation Center of Neuroregeneration, Nantong University, Nantong, China; 6 Shanghai Key Laboratory of Anesthesiology and Brain Functional Modulation, Translational Research Institute of Brain and Brain-Like Intelligence, Shanghai Fourth People’s Hospital Affiliated to Tongji University School of Medicine, Shanghai, China; 7 Department of Psychiatry, The Second Xiangya Hospital of Central South University, Changsha, Hunan China; 8 National Clinical Research Center for Mental Disorders, Changsha, Hunan China; 9 School of Health and Life Sciences, University of Health and Rehabilitation Sciences, Qingdao, Shandong China

**Keywords:** Chronic restraint stress, Blood–brain barrier, T helper 17 cells, Neuroinflammation, Depressive-like behaviour

## Abstract

**Background:**

Depression is a recurrent and devastating mental disease that is highly prevalent worldwide. Prolonged exposure to stressful events or a stressful environment is detrimental to mental health. In recent years, an inflammatory hypothesis has been implicated in the pathogenesis of stress-induced depression. However, less attention has been given to the initial phases, when a series of stress reactions and immune responses are initiated. Peripheral CD4^+^ T cells have been reported as the major contributors to the occurrence of mental disorders. Chronic stress exposure-evoked release of cytokines can promote the differentiation of peripheral CD4^+^ cells into various phenotypes. Among them, Th17 cells have attracted much attention due to their high pathogenic potential in central nervous system (CNS) diseases. Thus, we intended to determine the crucial role of CD4^+^ Th17 cells in the development of specific subtypes of depression and unravel the underpinnings of their pathogenetic effect.

**Methods:**

In the present research, a daily 6-h restraint stress paradigm was employed in rats for 28 successive days to mimic the repeated mild and predictable, but inevitable environmental stress in our daily lives. Then, depressive-like symptoms, brain–blood barrier (BBB) permeability, neuroinflammation, and the differentiation and functional changes of CD4^+^ cells were investigated.

**Results:**

We noticed that restrained rats showed significant depressive-like symptoms, concomitant BBB disruption and neuroinflammation in the dorsal striatum (DS). We further observed a time-dependent increase in thymus- and spleen-derived naïve CD4^+^ T cells, as well as the aggregation of inflammatory Th17 cells in the DS during the period of chronic restraint stress (CRS) exposure. Moreover, increased Th17-derived cytokines in the brain can further impair the BBB integrity, thus allowing more immune cells and cytokines to gain easy access to the CNS. Our findings suggested that, through a complex cascade of events, peripheral immune responses were propagated to the CNS, and gradually exacerbated depressive-like symptoms. Furthermore, inhibiting the differentiation and function of CD4^+^ T cells with SR1001 in the early stages of CRS exposure ameliorated CRS-induced depressive-like behaviour and the inflammatory response.

**Conclusions:**

Our data demonstrated that inflammatory Th17 cells were pivotal in accelerating the onset and exacerbation of depressive symptoms in CRS-exposed rats. This subtype of CD4^+^ T cells may be a promising therapeutic target for the early treatment of stress-induced depression.

**Supplementary Information:**

The online version contains supplementary material available at 10.1186/s12974-022-02543-6.

## Introduction

Depression is a recurrent and devastating mental disease that is highly prevalent worldwide [1]. Prolonged exposure to stress increases vulnerability to mood disorders, including depression [2, 3]. Currently, city living may lead to various social and environmental stresses that are detrimental to mental health. Mounting evidence has demonstrated the correlation between predictable but inevitable stressors, such as confined working or living spaces, and the onset of depression [4–7]. However, the exact mechanism of how such a stressful event affects the central nervous system (CNS) remains unclear.

Recently, immune dysfunction in the CNS has been implicated in the pathogenesis of major depression [8, 9]. Our previous findings and those of others indicate that the severity of depression is associated with the secretion level of circulating cytokines and chemokines, which compromise the blood–brain barrier (BBB) and then activate central immune responses [10–13]. Once the overactivated neuroimmune response exceeds the resolving capacity of resident immune cells in the brain, psychiatric disorders develop. Nonetheless, less attention has been given to the initial phases of the disease. A recent clinical study strongly suggested that peripheral T helper (Th) cells can be a potential biomarker for the early prediction of depression [14]. Moreover, peripheral CD4^+^ T cells have been reported as the major contributors to the occurrence of mental disorders [15, 16]. These cells are susceptible to the changes in the cytokine milieu. Chronic stress exposure-evoked release of cytokines can promote the differentiation of peripheral CD4^+^ cells into various phenotypes [17]. Among them, Th17 cells have attracted much attention due to their high pathogenic potential in CNS diseases [18, 19]. Th17 cells are increased in mice exhibiting learned helplessness. Additionally, mice receiving Th17-cell administration display obvious depressive-like behaviours [16, 20]. All this evidence indicates a crucial role of Th17 cells in the pathogenesis of stress-induced depression. Therefore, we hypothesized that inflammatory CD4^+^ Th17 cells may be involved in accelerating the development of depression and exacerbating disease symptoms. Several key issues, such as how Th17 cells are recruited into the CNS and how they impact disease progression, still need to be addressed.

Increasing evidence from clinical studies indicates that the severity of depression in unmedicated patients is associated not only with increased levels of proinflammatory cytokines, but also with the structural and functional alterations in the dorsal part of the striatum [21–23]. Aberrant activity in the dorsal striatum (DS) has been implicated in the core symptoms of depression, such as motivational anhedonia and psychomotor retardation. In addition, our preliminary research suggested that the DS showed the most significant BBB disruption among the tested brain regions (see Fig. 2c and Additional file 1: Fig. S1) after chronic restraint stress (CRS) exposure. CRS represents a continuous mild and predictable, but inevitable stress. It can partially mimic the daily repetitive exposure in stressful jobs and living environments. It is an ideal model for studying the underlying mechanisms of prolonged stress exposure-induced depression. Thus, in the present research, we placed particular emphasis on the correlation between pathological changes in the DS and stress-induced depressive-like symptoms. We intended to determine the crucial role of CD4^+^ Th17 cells in the development of specific subtypes of depression and unravel the underpinnings of their pathogenetic effect. We ultimately attempted to provide novel insights into finding potential therapeutic targets to treat chronic stress-induced depression.

## Materials and methods

### Animals and grouping

In total, ninety-six, 6- to 8-week-old male Wistar rats weighing 180–200 g at the beginning of the present research were purchased from Vital River (Beijing, China). All rats were housed in a temperature (22 ± 2 ℃) and humidity (60%) controlled room with a standard light–dark cycle (12 h:12 h). Rats were provided free access to water but restricted food access (15 g/day/rat). Rats were randomly assigned to corresponding groups to receive sham, CRS or CRS + SR1001 treatment. SR1001 (C_15_H_13_F_6_N_3_O_4_S_2_) (CAS No.:1335106-03-0) was purchased from Med Chem Express (MCE, New Jersey, USA). Rats in the CRS + SR1001 group received a daily intraperitoneal (IP) injection of SR1001 at a does of 25 mg/kg. All procedures were approved by the Institutional Animal Care and Use Committee at the Hunan University of Chinese Medicine and conformed to the National Institutes of Health Guide for the Care and Use of Laboratory Animals.

### Chronic restraint stress

Rats in the CRS groups received behavioural restraint 6 h per day (from 10:00 to 16:00) for 7, 14 and 24 successive days. Rats were placed in well-ventilated Plexiglass tubes (length, 16 cm and inner diameter, 6 cm) without food and water supply. During the restraint phase, control animals were handled for 5 min and kept in their home cage without food and water supply. After CRS exposure, restrained animals were returned to their home cage.

### Behavioural tests

#### Sucrose preference test (SPT)

Before testing, rats were acclimated to drinking water and sucrose solution. Two bottles of equivalent liquid were provided to the animals. One contained 1% sucrose, and the other contained water. The positions of the two bottles were changed every day during acclimation. On the testing day, two bottles were presented in their home cage for 24 h. Finally, fluid remains were weighed to calculate liquid consumption. Sucrose preference was calculated as follows: sucrose preference rate = sucrose consumption (g)/[sucrose consumption (g) + water consumption (g)] × 100%.

#### Forced swim test (FST)

Rats were placed separately in a glass cylinder (50 cm in height and 30 cm in diameter) containing 35 cm of water (24 ± 1 ℃). Water was renewed before each test. Each rat was forced to swim for 5 min and returned to their home cage immediately. The test was recorded by a camera and analysed later by two well-trained observers who were blinded to the grouping. A rat was judged to be immobile as it floated in an upright position and made few necessary movements to hold its head above the surface of water. The duration of immobility was recorded during the 5 min test.

#### Novelty-suppressed feeding test (NSFT)

Twenty-four hours before the test, rats were food-deprived. Rats were put into a novel open-field box (50 × 40 × 30 cm) from the same corner. Equal-sized food pellets were placed in the centre of the box. The latency to consume chow was manually recorded during a session of 6 min.

### Blood–brain barrier permeability

Fluorescein sodium injection (C_20_H_10_Na_2_O_5_, 3 ml:0.6 g) was purchased from Guangxi Wuzhou Pharmaceutical Co., Ltd (China). Rats were subjected to IP injection of 200 µl sodium fluorescein. Two hours later, the animals were anaesthetized with 1% pentobarbital. Blood samples were collected via cardiac puncture before transcardial perfusion with 0.9% saline. Subsequently, brains were removed, and the DLS and DMS were collected. Then, tissues were homogenized in PBS and centrifuged at 4 ℃ (14,000 *g*) for 5 min. Subsequently, 500 μl of the supernatant was mixed with 500 μl of 15% trichloro-acetic acid (TCA) and centrifuged (4 ℃, 1000 *g*) for 10 min. After that, 500 μl of the supernatant was mixed with 125 μl 5 M NaOH, and the fluorescence of the mixture was transferred to a 96-well plate and detected by a microplate reader (Thermo Fisher, USA) (excitation 485 nm, emission 530 nm). Blood samples were centrifuged at 4 ℃ (3000 rpm) for 10 min to obtain serum. Serum was mixed with 15% TCA (1:10) first and then mixed with 5 M NaOH (4:1). The fluorescence of the mixture was detected after dilution in PBS (1:500). The BBB permeability was presented as: [tissue fluorescence/serum fluorescence]^CRS^/[tissue fluorescence/serum fluorescence]^Cont^.

### Sample collection

After behavioural testing, animals were killed by deep anaesthesia (1% pentobarbital; IP). Blood samples were collected via cardiac puncture before transcardial perfusion with 0.9% ice-cold saline. Brain samples for immunofluorescence were collected from rats that subsequently received transcardial perfusion with 4% paraformaldehyde (PFA). Brain samples for WB and ELISA were quickly removed, and the DLS and DMS (see Fig. 2a) were dissected on ice. Samples for flow cytometric analysis were collected without specific processing before testing.

### Immunofluorescence and quantification analysis

Brains were postfixed overnight in 4% PFA before being dehydrated with sucrose solution (10%, 20%, 30%) for 3 days at 4 ℃. Brains were cut into 50-μm-thick sections in the coronal plane. Sections containing DLS and DMS were rinsed with 0.5% Triton X-100 (Solarbio, China) for 5 min, and blocked with 5% BSA (Sangon Biotech, China) for 1 h at room temperature. Then, the sections were incubated with mouse anti-CD31 (ab64543, 1:200, Abcam, UK), rabbit anti-claudin 5 (ab15106, 1:200, Abcam, UK), chicken anti-GFAP (ab4674, 1:2000, Abcam, UK), rabbit anti-Iba1 (ab153696, 1:500, Abcam, UK), rabbit anti-IL17A (A0688, 1:50, Abclonal) and mouse anti-CD4 (67786-1-lg,1:200, Proteintech) antibodies for 48 h at 4 ℃ in the dark. Then, the sections were incubated with Alexa-568 donkey anti-rabbit IgG (ab175470, 1:1000, Abcam, UK), Alexa-488 donkey anti-mouse IgG (ab150105, 1:1000, Abcam, UK), and Alexa-594 goat anti-chicken IgG (ab150172, 1:1000, Abcam, UK) for 2 h at room temperature. Finally, sections were mounted on slides with Fluoroshield™ with DAPI (F6057, Sigma-Aldrich, USA). Images were captured with a Nikon A1R HD25 confocal microscope system (Nikon, Japan). Immunofluorescence quantification was performed as previously described [24] and detailed in the Additional file 1.

### Western blotting

Tissue was ground in 500 μl RIPA buffer mixed with protease inhibitor cocktail tablets (cOmplete ULTRA Tablets, Roche, Germany) on ice, followed by centrifugation at 4 ℃ (14,000 rpm) for 10 min. The supernatant was collected as protein and stored at − 80 ℃ for the next step. The protein concentration was ascertained by a commercial BCA kit (MultiSciences, China). Electrophoresis on SDS–PAGE gels (CW2384, CWBIO, China) was used to separate the protein extracts that were transferred onto polyvinylidene difluoride membranes (0.22 μm, Merck Millipore, Germany). The membrane was incubated overnight with the following primary antibodies: anti-claudin-5 (ab15106, 1:1000, Abcam, UK), anti-MMP2 (ab92536, 1:1000, Abcam, UK), anti-MMP9 (ab38898, 1:1000, Abcam, UK), anti-β-actin (1:1000, Proteintech, China) and anti-GAPDH (1:1000, Proteintech, China). The immunoreactive bands were visualized using goat anti-mouse (1:8000, Proteintech, China) and goat anti-rabbit (1:8000, Proteintech, China) secondary antibodies and ECL Chemiluminescence HRP substrate (WBKLS0500, Merck Millipore, Germany) followed by autoradiography. The intensity of the blots was analysed with ImageJ.

### Flow cytometric analysis

At the indicated time points, the spleen, thymus and DS were quickly dissected on ice, and rinsed with ice-cold 0.9% saline. Tissues were cut into pieces in 10-cm dishes added to 0.9% saline.

Spleen and thymus tissue pieces were ground in a MagicFilter (Bozhen Tech, B160203-01) to collect the cell suspension. Then, the cell suspension was centrifuged at 500 × *g* for 5 min. Cell staining buffer (Biolegend, 420201) was added to resuspend the cells. Then, 100 μl of the cell suspension was removed into a tube and mixed with 1 μl of the zombie Aqua Fixable Viability kit (Biolegend, 423101) before being incubated for 15 min in the dark. Subsequently, antibodies (APC anti-rat CD4, FITC anti-rat CD3, PE anti-rat CD8a; Biolegend) were added and incubated in the dark for 20 min. After that, the samples were centrifuged at 350 *g* for 5 min, and 300 μl of cell staining buffer was added to resuspend the cells. The prepared spleen and thymus samples were analysed with a flow cytometer (BD, Diva 6.1.3) and FlowJo software (10.5.3).

Moreover, striatal tissues were ground to collect the cell suspension. Cell stimulation cocktail (plus protein transport inhibitors, 500x) (eBiosciences, 00-4975-93) and eBioscience™ Intracellular Fixation & Permeabilization Buffer Set (eBiosciences, 88-8824-00) were used to collect the cell suspension and incubated with antibodies (anti-Rt CD3, anti-Rt CD4, anti-Mo/Rt IL-17A, eBiosciences) for 30 min in the dark. Samples were washed with 1 ml PBS and centrifuged at 400 × g for 5 min, and 350 μl PBS was added to resuspend cells, which were then analysed with another flow cytometer (Beckman, A00-1–1102) and FlowJo software (cytexpert 2.0). The gating strategy and fluorescence-minus-one (FMO) control are detailed in Additional file 1: Fig. S3.

### ELISA

Tissues and serum levels of VEGF, TNF-α, IL-1β, IL-6, IL-17 and IL-22 were evaluated with commercially available high-sensitivity enzyme-linked immunosorbent assay (ELISA) kits (MultiSciences, China) according to the manufacturer’s instructions. Absorbance at 450 nm and 570 nm was measured by a microplate reader (ELx808, Biotek, USA).

### Statistical analysis

All experiments were replicated twice. Data were analysed with GraphPad Prism version 7.0 (GraphPad, San Diego, CA, USA). Data are expressed as the means ± SEM. Measures were analysed using a *t*-test or one-way analysis of variance (ANOVA) where statistically appropriate. ANOVA was followed with LSD post hoc multiple comparisons. A *p* value < 0.05 was defined as statistically significant.

## Results

### CRS results in obvious depressive-like symptoms in rats

**Fig. 1:**
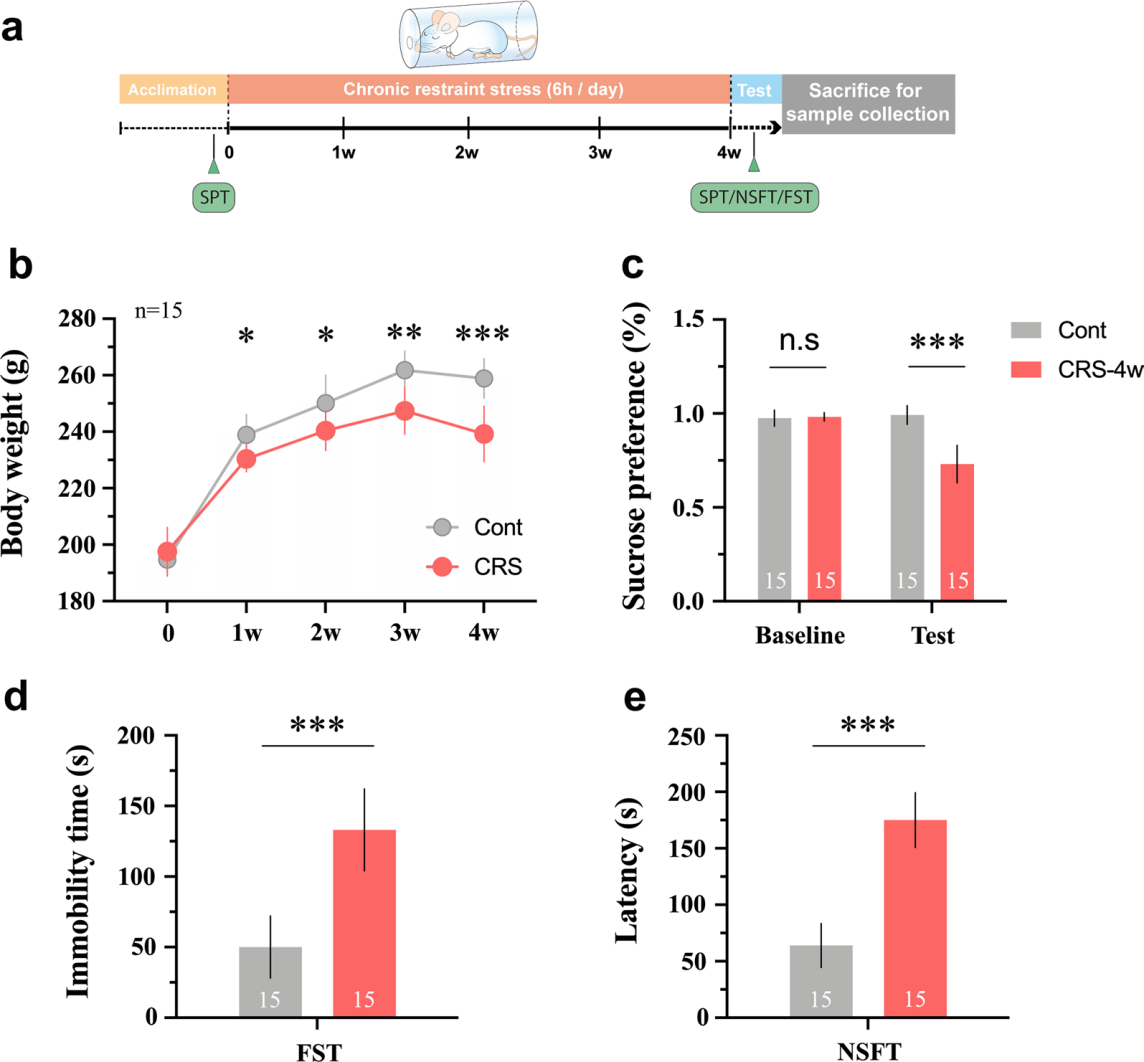
CRS results in obvious depressive-like symptoms in rats. **a** The experimental scheme of CRS exposure and behavioural tests; **b** body weight changes during the experiment; compared to control group, rats in CRS group shows **c** decreased sucrose consumption in SPT, **d** increased immobility in FST and **e** longer latency to eat chow in NSFT, after 28 days of chronic stress exposure. Data are expressed as mean ± SD, Student’s *t*-test, *p < 0.05, **p < 0.01, ***p < 0.001, compared with control

Figure 1a shows the experimental scheme of CRS exposure and behavioural tests. Rats were subjected to a 28 days of restraint stress. Then, CRS-induced depressive-like behaviours were evaluated. As shown in Fig. 1b, the increase in body weight was significantly inhibited during CRS exposure, compared with control rats (1 w, *p* < 0.05; 2 w, *p* < 0.05; 3 w, *p* < 0.01; 4 w, *p* < 0.001;). The SPT was conducted to evaluate anhedonia, a key depressive-like behaviour in experimental animals. The data shown in Fig. 1c indicate that the baseline sucrose consumption in animals in both the control and CRS groups was almost the same, whereas rats in the CRS group showed an obvious decrease in sucrose intake after CRS exposure (*p* < 0.01). Moreover, the FST was used to assess the severity of behavioural despair. CRS-exposed rats exhibited increased immobility compared with rats in the control group (Fig. 1d, *p* < 0.001). Furthermore, NSFT is employed to evaluate the conflict behaviour between novel environment-induced anxiety that delays food intake and the desire for feeding. The latency to take food is a sensitive parameter for detecting the impact on appetite. Rats in the CRS group spent a significantly longer time initially approaching and eating the chow located in the centre of the box than those in the control group (Fig. 1e, *p* < 0.001).

### CRS leads to BBB disruption in the DLS and DMS

**Fig. 2:**
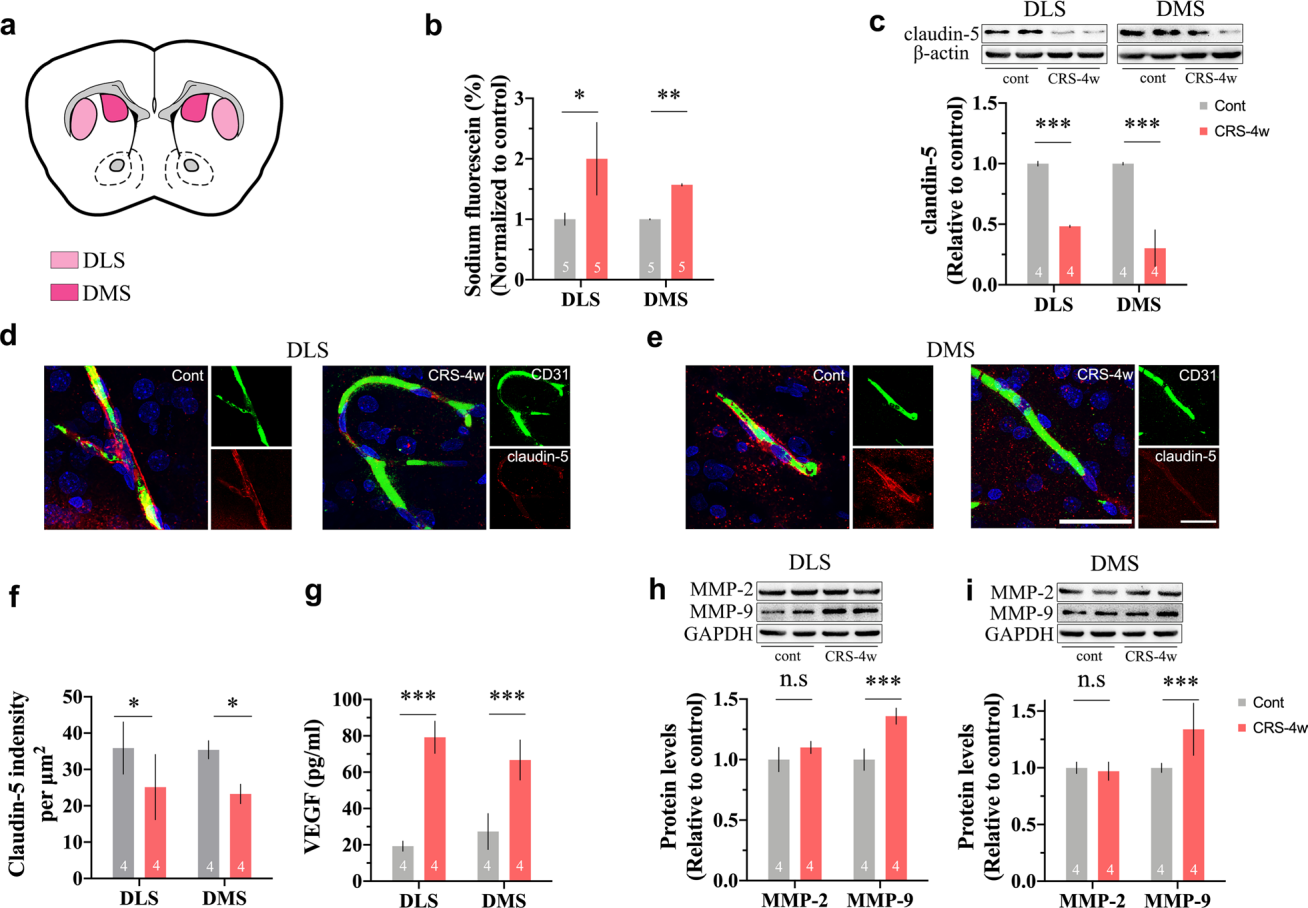
CRS leads to BBB disruption in the DLS and DMS. **a** Tissue dissection sites; **b** CRS increased fluorescein sodium leakage into the DLS and DMS; **c** the expression of TJs protein claudin-5 is significantly decreased in the DLS and DMS of rats exposed to CRS; **d** and **e** representative confocal images of the expression changes of claudin-5 in the BBB; **f** quantification of claudin-5 immunoreactivity in the DLS and DMS; **g**–**i** the expression changes in vascular permeability factors. The expression of VEGF and MMP-9, but not MMP-2, are increased in the DLS and DMS; scale bar, 25 μm. Data are expressed as mean ± SD, Student’s *t*-test, *p < 0.05, **p < 0.01, ***p < 0.001, compared with control

BBB permeability was evaluated by the ratio of fluorescein sodium leaked into the brain parenchyma. As shown in Fig. 2b, CRS exposure aggravated BBB leakage in both the DLS (*p* < 0.05) and DMS (*p* < 0.05). Moreover, the expression levels of claudin-5 in the DLS (*p* < 0.001) and DMS (*p* < 0.001) were significantly reduced in the CRS group (Fig. 2c). Then, we determined the pathological changes in the BBB. CD31 is a well-recognized marker for ECs. As shown in Fig. 2d and e, we detected a reduction in claudin-5 protein in the DLS and DMS of rats exposed to CRS by examining its colocalization with CD31. The immunoreactivity of claudin-5 in the DLS (*p* < 0.05) and DMS (*p* < 0.05) was obviously decreased (Fig. 2f). Furthermore, the expression changes in vascular permeability factors were determined (Fig. 2 g–i). The release of VEGF was highly increased in both the DLS (*p* < 0.001) and DMS (*p* < 0.001). We noticed that the expression levels of MMP-9 but not MMP-2 were obviously increased in both the DLS (*p* < 0.001) and DMS (*p* < 0.05).

### CRS causes an inflammatory response in the CNS

**Fig. 3:**
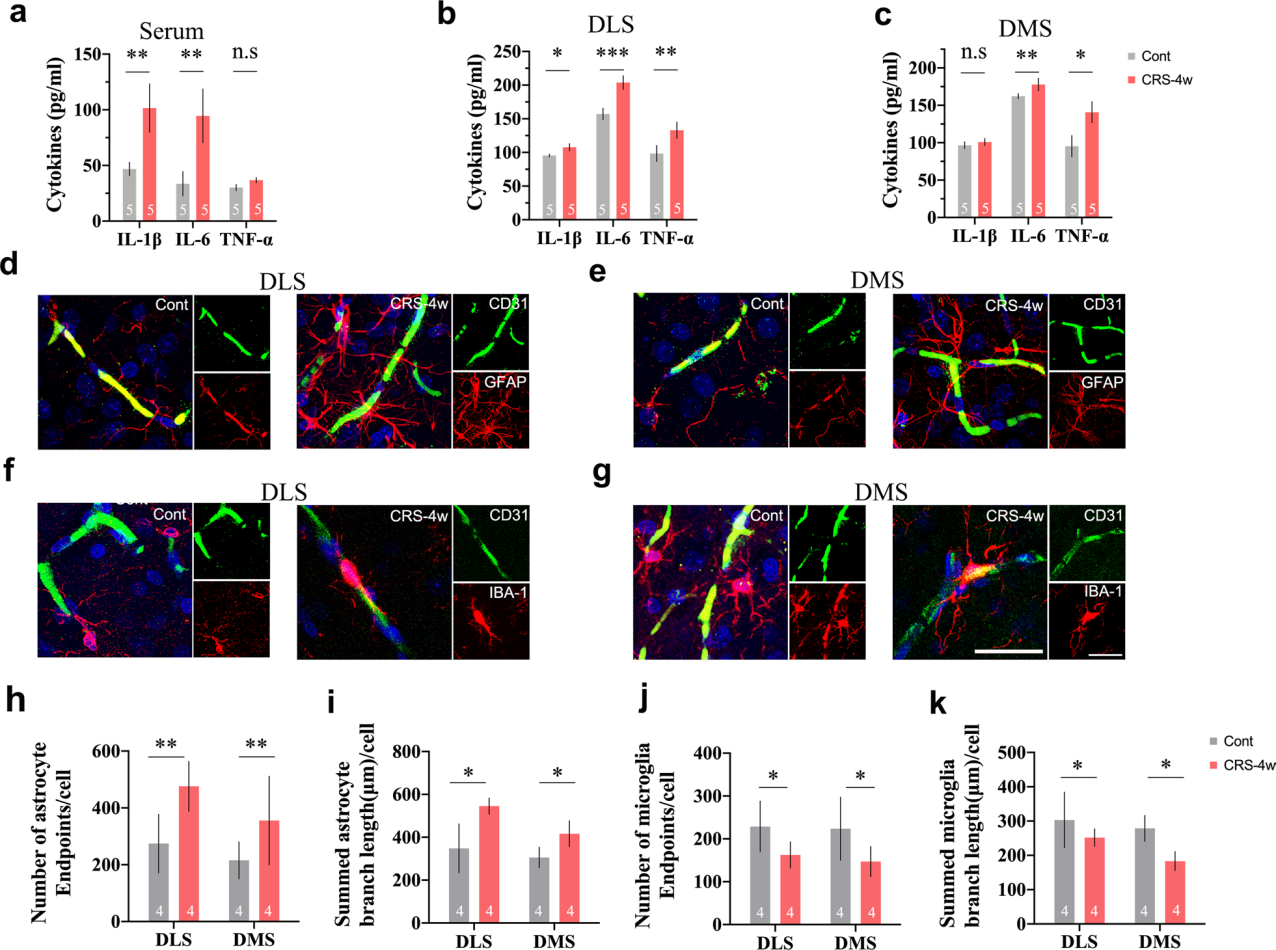
CRS causes an inflammatory response in the CNS. **a**–**c** The expression levels of major cytokines, such as IL-1β, IL-6 and TNF-α are elevated in serum, the DLS and DMS of CRS rats; **d** and **e**) CRS-induced morphological changes in astrocytes near the BBB in the DLS and DMS; **f** and **g** CRS-induced morphological changes in microglia nearing the BBB in the DLS and DMS; **h**–**k** quantification of morphological changes in astrocytes and microglia. Scale bar, 25 μm. Data are expressed as mean ± SD, Student’s *t*-test, *p < 0.05, **p < 0.01, ***p < 0.001, compared with control

To determine the CRS-induced impact on the CNS, we assessed changes in cytokines and glial cells in the DLS and DMS. As shown in Fig. 3a–c, the expression levels of major inflammatory cytokines, such as IL-1β (*p* < 0.01) and IL-6 (*p* < 0.01) in serum; IL-1β (*p* < 0.05), IL-6 (*p* < 0.001) and TNF-α (*p* < 0.01) in the DLS; and IL-6 (*p* < 0.05) and TNF-α (*p* < 0.01) in the DMS of rats exposed to CRS, were significantly elevated. We further investigated whether CRS resulted in morphological alterations in astrocytes and microglia near the BBB with specific markers such as glial fibrillary acidic protein (GFAP) and ionized calcium-binding adapter molecule-1 (Iba-1). We noticed that reactive astrogliosis occurred in the DLS and DMS of rats exposed to CRS (Fig. 3d, e). The number of astrocyte endpoints and summed branch length were significantly increased (Fig. 3h, i). Furthermore, we observed ramified microglia in the DLS and DMS of rats in the control group. However, microglia in the DLS and DMS of rats exposed to CRS exerted a deramified appearance with an enlarged soma (Fig. 3f, g). The number of microglial endpoints and summed branch length were significantly decreased (Fig. 3j, k). These findings indicated that astrocytes and microglia were obviously activated in the DLS and DMS after CRS exposure.

### CRS mobilizes peripheral CD4^+^ Th17 cells into the brain in the initial phases of depression

**Fig. 4:**
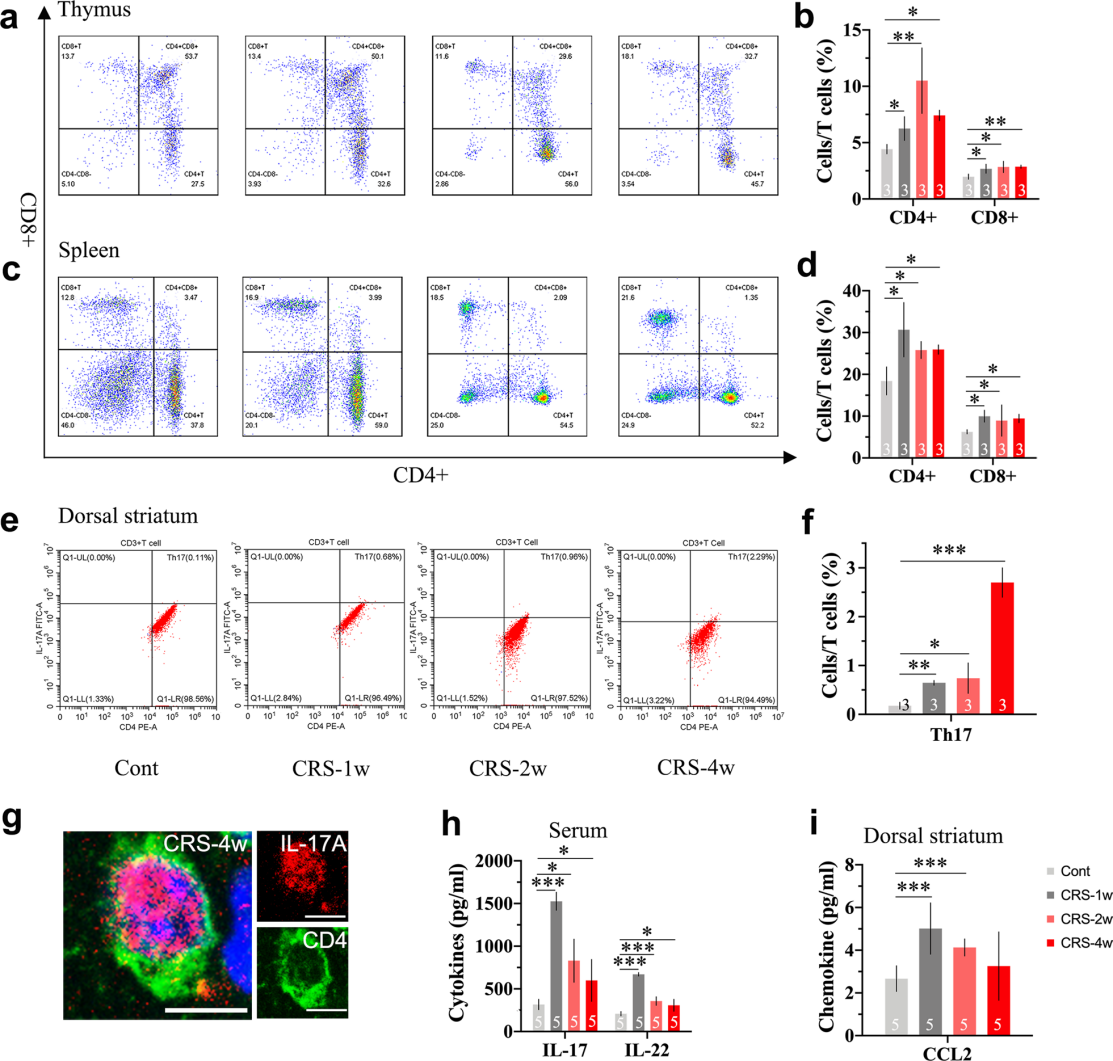
CRS mobilizes peripheral CD4^+^ Th17 cells into the brain in the early phases of stress exposure. **a**–**d** Representative flow cytometric analysis and statistical result of CD4^+^ and CD8^+^ T cells contents in the thymus and spleen; **e**, **f** representative flow cytometric analysis and statistical result of CD4^+^ Th17 cells contents in the dorsal striatum; **g** representative confocal images of CD4^+^IL17A^+^ T cells; the expression levels of chemokine **h** CCL2 in serum; cytokines; **i** IL-17; and IL-22 in the dorsal striatum. Scale bar, 20 μm. Data are expressed as mean ± SD, Student’s *t*-test, *p < 0.05, **p < 0.01, ***p < 0.001, compared with control

To determine where does the inflammation comes from, we focused on the early phases of CRS exposure. The thymus and spleen are critical peripheral immune organs that play pivotal roles in initiating and propagating immune responses. Flow cytometric analysis was conducted to identify CD4^+^ and CD8^+^ T cells in the thymus and spleen. As shown in Fig. 4a–d, the contents of CD4^+^ and CD8^+^ T cells in the spleen and thymus were increased throughout the period of CRS exposure. Notably, the contents of naïve CD4^+^ cells peaked in the first week in the spleen (*p* < 0.05) and in the second week in the thymus (*p* < 0.01). Moreover, we noticed that the contents of Th17 cells in the DS were significantly increased throughout the period of CRS exposure (Fig. 4e, f). Representative confocal images showed that CD4 and IL-17A were colocalized on T cells (Fig. 4g). The number and immunoreactivity of CD4 and IL-17A costained T cells also increased with disease progression (Additional file 1: Fig. S2). The expression level of the chemokine CCL2 in the circulation reached its highest level in the first week of CRS exposure and returned to normal in the fourth week (Fig. 4h). Moreover, the expression levels of IL-17 and IL-22 in the DS rose sharply in the first week of CRS exposure and became flat later, although the values remained higher than those in the control group (Fig. 4i). Our data suggested that peripheral Th17 cells were recruited into the CNS in the early phases of CRS exposure.

### Inhibiting CD4^+^ T-cell differentiation into pathogenic Th17 cells is effective in ameliorating CRS-induced depressive-like behaviour

**Fig. 5:**
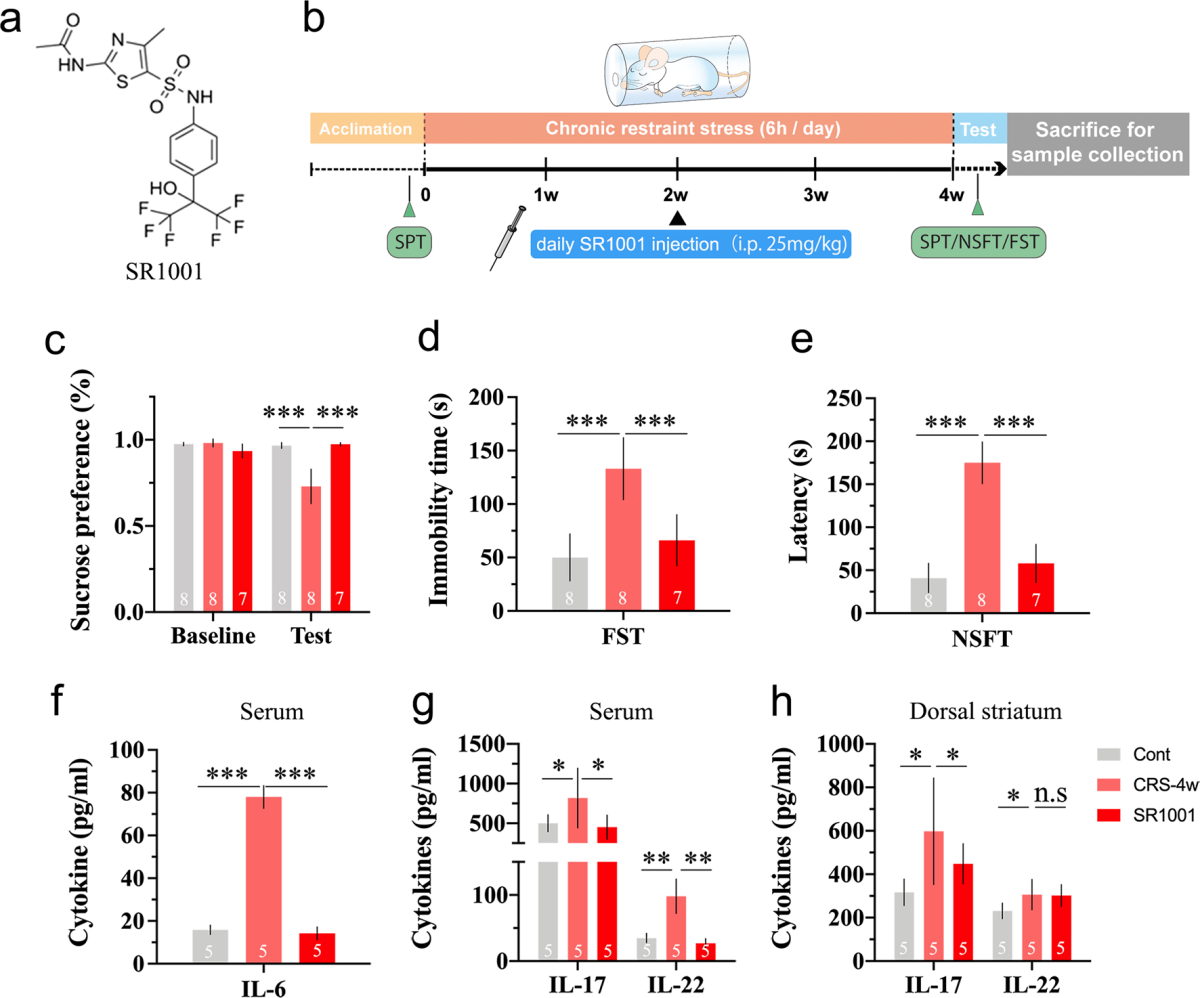
Inhibiting CD4^+^ T-cell differentiation into pathogenic Th17 cells is effective in ameliorating CRS-induced depressive-like behaviour. **a** Chemical structure of SR1001; **b** experimental scheme of SR1001 treatment; compared to CRS group, rats showed increased sucrose consumption in SPT (**c**), decreased immobility in FST (**d**) and shorter latency to eat chow in NSFT (**e**) after SR1001 treatment; the expression levels of IL-6 in serum (**f**); IL-17 and IL-22 in serum (**g**) and dorsal striatum (**h**) were fully or partially reversed after SR1001 treatment. Data are expressed as mean ± SD, ANOVA followed with LSD post hoc, *p < 0.05, **p < 0.01, ***p < 0.001, compared with CRS group

To further determine the role of inflammatory Th17 cells in the development of CRS-induced depressive-like symptoms, SR1001 was employed to verify the therapeutic effects of interfering with Th17 cells. SR1001 is a high-affinity synthetic ligand that specifically binds to both RORα and RORγt domains to inhibit Th17-cell differentiation and function. Figure 5a shows the chemical structure of SR1001, and Fig. 5b illustrates the experimental scheme. As shown in Fig. 5c–e, SR1001 treatment significantly ameliorated CRS-induced depressive-like behaviour in the SPT (*p* < 0.001), FST (*p* < 0.001) and NSFT (*p* < 0.001) compared to rats only exposed to CRS. Moreover, as shown in Fig. 5f and g, the expression levels of IL-6 (*p* < 0.001), and the levels of IL-17 (*p* < 0.05) and IL-22 (*p* < 0.01) in serum were obviously decreased after SR1001 treatment. Notably, the expression levels of IL-17 (*p* < 0.05) and IL-22 in the DS were either moderately decreased or unchanged (Fig. 5h). Furthermore, the activation of astrocytes and microglia in the DLS and DMS was suppressed after SR1001 treatment (Additional file 1: Fig. S4).

## Discussion

Prolonged exposure to stressful events or a stressful environment is detrimental to mental health [25]. In recent years, an inflammatory hypothesis has been implicated in the pathogenesis of stress-induced depression. However, as we mentioned above, less attention has been given to the early phases of disease development, when a series of stress reactions and immune responses are just initiated. In our present research, we investigated the pathogenetic effect of CD4^+^ Th17 cells in the pathogenesis of chronic stress-induced depression and intended to unravel the underlying mechanisms.

In our daily lives, social and physical environments have profound impacts on our brains. Our brain in turn responds to stressors with either physiological or behavioural adaptation [26]. Although moderate stress is helpful for learning and memory, persistent or chronic stress exposure can precipitate or exacerbate mental disorders [6, 27]. Living in a modern metropolis, many people suffer from limited working or living space. Such environmental stress is relatively mild and predictable but inevitable. The CRS model ideally mimics the key features of this type of stressor and has been used as a major experimental model of depression [28, 29]. Core symptoms of depression include anhedonia, depressed mood, despair, cognitive impairment, etc. [30]. Consistent with the findings of previous studies, rats that suffered 28 days of CRS exposure showed obvious depressive-like symptoms. The body weight increases in rats exposed to CRS was significantly suppressed throughout the experiment. Considering that food and water were deprived as well in the control group during the 6-h behaviour restraint, the suppressed increase in body weight suggested an appetite reduction in rats exposed to CRS. Moreover, rats exposed to CRS spent more time overcoming novel environment-induced anxiety to eat food located in the centre of an open-field box. The increased feeding latency is also a sensitive parameter for assessing appetite. In addition, rats exposed to CRS exhibited less sucrose consumption, an indicator of anhedonia, and increased immobility, a parameter for evaluating behavioural despair, than control rats.

The striatum, a subcortical region of the forebrain, lies at the central interface of the motor and reward systems. It receives inputs from multiple brain regions and converges sensory, emotional, and cognitive information to guide behavioural output, including motivation, reward perception, reinforcement, decision-making, and motor and action planning [31, 32]. The striatum can be divided into a dorsal and a ventral part. The ventral part of the striatum is also known as the nucleus accumbens (Nac), which is implicated in reward processing and reinforcement [33]. Moreover, the dorsomedial striatum (DMS) and dorsolateral striatum (DLS) constitute the DS. Generally, DMS regulates goal-directed actions, whereas DLS determines habitual responses [34–36]. Goal-directed behaviour is associated with the value of the outcome. Once the outcome of a behaviour is perceived as meaningless or vapid, motivation-related anhedonia emerges. Habitual behaviour relies on previous experience, particularly the memory of antecedent stimuli. Impaired cognition results in dysfunction in normal memory retrieval, thereby disrupting the ability to plan and take a proper action. In short, both functional subregions of the DS are pivotal in regulating value- and habit-based decision-making and perceiving the reward associations underlying these behaviours.

It has been recognized that stress-induced depression is accompanied by an inflammatory response in both the periphery and the CNS. Nonetheless, the sources of inflammatory factors are diverse [37]. In our study, we noticed that the levels of proinflammatory cytokines, such as interleukin (IL)-1β, IL-6 and tumour necrosis factor (TNF)-α were significantly increased in serum and brain tissues after CRS exposure. Abundant experimental evidence suggests that the BBB is compromised after repeated stress exposure [11, 38, 39]. The BBB protects the brain from potentially harmful toxins and pathogens in the periphery [40]. It is a physical barrier consisting of endothelial cells (ECs), pericytes, astrocytes and tight junctions (TJs). Chronic stress first evokes the release of proinflammatory cytokines into the circulation. Blood-borne cytokines and chemokines then accumulate in the brain and subsequently assault the TJs that tightly seal the gaps between ECs. Once the barrier function of the BBB is impaired, proinflammatory factors infiltrate the brain and activate the innate immune system in the CNS. Dysregulated neuroinflammation leads to the occurrence of depression [41]. The breakdown of the BBB is mainly due to the disruption of TJs, such as claudin-5 [42, 43]. In our study, the contents of IL-1β and IL-6 were obviously higher in the circulation of rats exposed to CRS. Meanwhile, we observed an increased fluorescein sodium leakage into the DLS and DMS, which reflected BBB leakage. Infiltrated cytokines subsequently evoked immune responses in the CNS and led to the activation of astrocytes and microglia near the BBB. Reactive astrocytes are a major source of various vascular permeability factors [44–47]. In line with previous findings in both clinical and experimental research [48, 49], we found that vascular endothelial growth factor (VEGF) levels were significantly elevated in restrained rats. As a pleiotropic growth factor, VEGF is responsible for multiple important physiological functions, particularly BBB permeability [44, 50, 51]. Moreover, several studies in ischaemic stroke, a neurological disease characterized by BBB impairment, suggest that the breakdown of claudin-5 can be attributed to VEGF-mediated matrix metalloproteinase (MMP) expression [52–55]. MMPs are zinc-containing endopeptidases that can remodel and degrade the extracellular matrix and TJ proteins surrounding ECs. MMP-2 and MMP-9 are the most studied gelatinases in the brain [56]. Stress is the leading cause of MMP-9 secretion in either patients or experimental animals [57, 58]. Consistently, we noticed a significant increase in MMP-9 expression but not MMP-2 in brain tissues of both the DLS and DMS. In addition, as shown in Fig. 3a and b, the contents of inflammation-related mediators were highly increased in the DLS and DMS. Microglia, the target as well as the source of proinflammatory cytokines, displayed a deramified shape with an enlarged soma in rats exposed to CRS. In the resting state, microglia are highly ramified and acutely sensitive to microenvironmental homeostasis within the CNS. Activated astrocytes and microglia respond rapidly to pathological stimuli with morphological and functional changes, thereby exacerbating neuroinflammation [40, 59]. Our findings indicate that CRS-induced depressive-like symptoms are closely correlated with astrocyte-derived VEGF/MMP-9 signalling-mediated BBB disruption and the subsequent outburst of neuroinflammation.

Breakdown of the BBB, accumulation of cytokines and activation of immune cells are key events in stress-induced depression. Nevertheless, the occurrence of depressive-like symptoms is a progressive process. Unveiling the mechanisms underlying the early-stage development of stress-induced depression, would contribute to the exploration of potential therapeutic targets for early treatment. Acute stress evokes transient responses in either the endocrine or immune system, whereas chronic stress ultimately results in permanent psychological or physical adaptations. In addition to the neuroendocrine system, the peripheral immune system is most vulnerable to stimuli in the early phases of stress exposure [60]. The thymus is a crucial lymphoid organ that is responsible for the proliferation and differentiation of T cells. Haematopoietic progenitors migrate into the thymus, where they become naïve T cells upon presentation with specific markers. Thymocytes can differentiate into CD8^+^ cytotoxic cells that clear damaged cells or CD4^+^ helper and regulatory cells coordinating the immune response [61]. The spleen is a peripheral immune organ where adaptive immunity is initiated [62]. Once primed, activated T cells leave the spleen and reenter the circulation. It has been suggested that chronic social defeat can lead to enhanced Th17 differentiation in the spleen [63]. As expected, we observed a time-dependent increase in thymus- and spleen-derived naïve CD4^+^ T cells in the present research, which peaked within the first two weeks. Highly inflammatory Th17 cells in the CNS can switch their surface signature from C–C chemokine receptor (CCR) 6 into CCR2 [64]. We noticed that chemokine (C–C motif) ligand (CCL) 2 secretion, which drives Th17-cell infiltration into the inflamed CNS, was highly increased in the first week of CRS and decreased over time. Intriguingly, the number of Th17 cells in the DS gradually increased and peaked at the fourth week of CRS. Meanwhile, we noticed that the change pattern of IL-17 and IL-22 expression in the DS was consistent with that of CCL2. IL-17 and IL-22 are major Th17-produced cytokines that generate tissue inflammation [18]. It is plausible that during initial phases, CCR2/CCL2 drives peripherally activated Th17 cells to penetrate the brain. In turn, IL-17 further impairs BBB integrity, thus allowing more immune cells and cytokines to gain easy access to the CNS [65, 66]. It has been suggested that astrocytes can respond to IL-17 by releasing mediators that promote tissue damage [67]. We inferred that this mechanism can partially account for the early BBB leakage in rats exposed to CRS. Our findings further suggest that Th17 cells are recruited into the CNS starting in the initial phase of CRS exposure. Through a complex cascade of events, the peripheral immune response is propagated into the CNS, and progressively exacerbates depressive-like symptoms.

Additionally, Th17 cells have been associated with depression in several experimental and clinical studies [16, 20, 68]. It is plausible that the recruitment of activated Th17 cells into the brain and the increased production of IL-17/22 are crucial in the onset and development of depressive-like symptoms. Therefore, we speculated that preventing the transformation of CD4^+^ T cells into inflammatory Th17 cells in the early phase of CRS exposure could contribute to the remission of depressive symptoms. The nuclear receptors retinoic acid receptor-related orphan receptors (RORs) α and γt play indispensable roles in the differentiation of naïve CD4^+^ T cells into effector Th17 cells. SR1001 is a high-affinity synthetic ligand that specifically binds to the ligand-binding domains of both RORα and RORγt [69]. Since RORα and RORγt are ligand-dependent transcription factors, SR1001 can induce a conformational alteration within the ligand-binding domain, thereby inhibiting the differentiation and function of Th17 cells. In our research, rats exposed to CRS received preventive injection of SR1001 before exposure to restraint stress. As expected, pretreatment with SR1001 was able to ameliorate stress-induced depressive-like behaviours. We noticed that peripheral expression levels of IL-6, IL-17 and IL-22 were significantly reduced in rats exposed to CRS pretreated with SR1001. It is noteworthy that, in the DS, the expression level of IL-17 was moderately decreased, whereas that of IL-22 was unchanged. This result indicates that SR1001 can partially reverse the production of Th17-derived IL-17 in the brain. This is largely because under pathological conditions, IL-17 can be secreted by several other cells in the CNS in addition to Th17 cells. Particularly, meningeal resident γδ17 T cells, which express RORγt as well, have been recently implicated in regulating anxiety-like behaviour and short-term memory via IL-17 signalling [70, 71]. Thus, further studies employing cell-type specific manipulation are required to elaborate the precise role of effector Th17 cells in the pathogenesis of stress-induced depression. Nonetheless, in the present study, early inhibition of Th17 differentiation and function showed certain potential in remitting stress-induced depressive-like symptoms.

## Conclusion

**Fig. 6:**
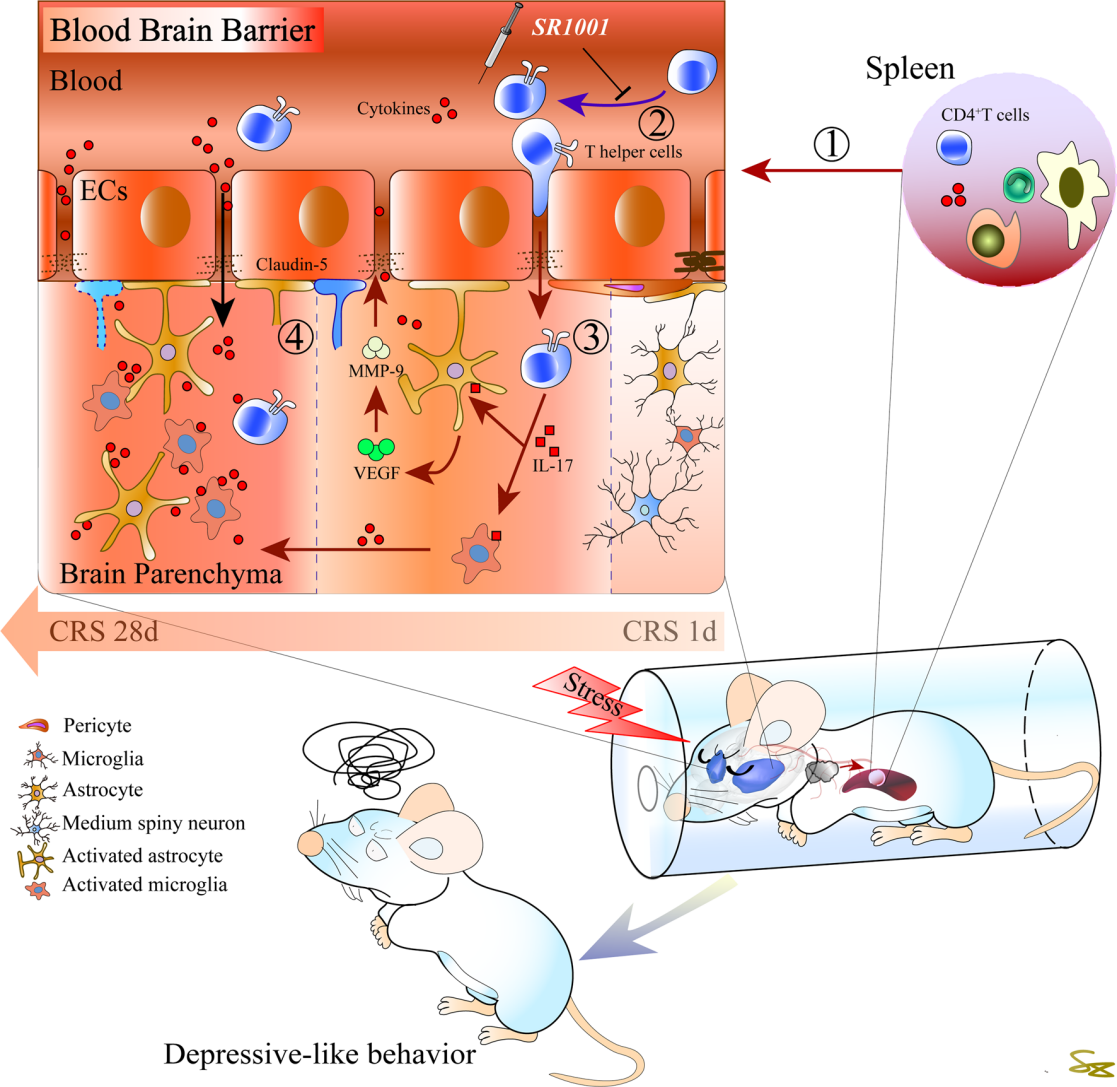
A schematic diagram illustrates the proposed mechanism. The recruitment of peripheral CD4^+^ Th17 cells into the CNS is closely associated with the development of CRS-induced depressive-like symptoms. Through a complex cascade of events, the peripheral immune response is propagated into the CNS through a leaky BBB, thereby progressively exacerbates neuroinflammation and disease symptoms

In summary, our findings indicate that the recruitment of peripheral CD4^+^ Th17 cells into the CNS is associated with the development of CRS-induced depressive-like symptoms. Through a complex cascade of events, the peripheral immune response is propagated into the CNS through a leaky BBB, thereby progressively exacerbating neuroinflammation and disease symptoms (illustrated in Fig. 6). Moreover, CD4^+^ Th17 cells may be a potential therapeutic target for the early treatment of chronic mild and predictable but inevitable stress-induced depressive-like symptoms.

## Supplementary Information

**Additional file 1: Fig. S1.** CRS leads to a decrease in claudin-5 expression in the (a) hippocampus; (b) prefrontal cortex (PFC) and (c) nucleus accumbens (Nac). Data are expressed as mean ± SD, Student’s t-test, *p < 0.05 compared with control. **Fig. S2.** CRS increases the accumulation of CD4+IL17A+ T cells in the dorsal striatum. Representative confocal images of CD4+IL17A+ T cells in the (a) DLS and (b) DMS; Cell counting of CD4+IL17A+ cells in the (c) DLS and (d) DMS. Scale bar, 20 μm. Data are expressed as mean ± SD, Student’s t-test, **p < 0.01 and ***p < 0.001 compared with control. **Fig. S3.** The gating strategy to identify Th17 cells. CD4+IL17A+ cells were selected from CD3+ T cells and gating with IL-17A staining and FMO control. **Fig. S4.** SR1001 prevents CRS-induced morphological changes in glial cells. SR1001 prevents CRS-induced morphological changes in (a) astrocytes and (b) microglia near the BBB in the DLS and DMS; (c) and (d) Quantification of morphological changes in astrocytes and microglia. Scale bar, 20 μm. Data are expressed as mean ± SD, Student’s t-test.

## Data Availability

The original data are available from the corresponding author upon request.

## Abbreviations

BBB: Blood–brain barrier
CCL: Chemokine (C–C motif) ligand
CCR: C–C chemokine receptor
CNS: Central nervous system
CRS: Chronic restraint stress
DS: Dorsal striatum
DLS: Dorsolateral striatum
DMS: Dorsomedial striatum
ECs: Endothelial cells
FST: Forced swim test
IL: Interleukin
MMPs: Matrix metalloproteinases
NSFT: Novelty-suppressed feeding test
ROR: Retinoic acid receptor-related orphan receptors
SPT: Sucrose preference test
Th cells: T helper cells
TNF: Tumour necrosis factor
TJs: Tight junctions
VEGF: Vascular endothelial growth factor
