# Supplementary material for: Chronic stress-induced depression requires the recruitment of peripheral Th17 cells into the brain
Source: J Neuroinflammation. 2022 Jul 14;19:186. doi: 10.1186/s12974-022-02543-6 (PMC9281140; doi:10.1186/s12974-022-02543-6)
Supplement: Supplementary file 1 — Additional file 1: Fig. S1. CRS leads to a decrease in claudin-5 expression in the (a) hippocampus; (b) prefrontal cortex (PFC) and (c) nucleus accumbens (Nac). Data are expressed as mean ± SD, Student’s t-test, *p < 0.05 compared with control. Fig. S2. CRS increases the accumulation of CD4+IL17A+ T cells in the dorsal striatum. Representative confocal images of CD4+IL17A+ T cells in the (a) DLS and (b) DMS; Cell counting of CD4+IL17A+ cells in the (c) DLS and (d) DMS. Scale bar, 20 μm. Data are expressed as mean ± SD, Student’s t-test, **p < 0.01 and ***p < 0.001 compared with control. Fig. S3. The gating strategy to identify Th17 cells. CD4+IL17A+ cells were selected from CD3+ T cells and gating with IL-17A staining and FMO control. Fig. S4. SR1001 prevents CRS-induced morphological changes in glial cells. SR1001 prevents CRS-induced morphological changes in (a) astrocytes and (b) microglia near the BBB in the DLS and DMS; (c) and (d) Quantification of morphological changes in astrocytes and microglia. Scale bar, 20 μm. Data are expressed as mean ± SD, Student’s t-test. [file 12974_2022_2543_MOESM1_ESM.docx]

Additional Materials for

**Chronic stress-induced depression** **requires the recruitment of peripheral Th17 cells into the brain**

*Zhuang Peng et al.*

Corresponding authors: zhezhe1106@163.com (Z. Shi); ytf0707@126.com (TF. Yuan); dfliao@hnucm.edu.cn (DF. Liao).

**This PDF file includes：**

Materials and Methods

Figs. S1 to S4

**Materials and Methods**

Flow cytometry

At the indicated time points, tissues were ground to collect the cell suspension. Cell stimulation cocktail (plus protein transport inhibitors, 500x) (eBiosciences,00-4975-93) and eBioscience™ Intracellular Fixation & Permeabilization Buffer Set (eBiosciences,88- 8824-00) were used to collect the cell suspension and incubated with antibodies (anti-Rt CD3, anti-Rt CD4, anti-Mo/Rt IL-17A, eBiosciences) 30 min in the dark. Samples were washed with 1 ml PBS and centrifuged at 400 g for 5 min, and 350 μl PBS was added to resuspend the cells. Then samples were analysed with a flow cytometer (Beckman, A00-1-1102) and FlowJo software (cytexpert 2.0).

Western Blotting

Tissue was ground in 500 μl RIPA buffer mixed with protease inhibitor cocktail tablets (cOmplete ULTRA Tablets, Roche, German) on ice, followed by centrifugation at 4℃ (14000 rpm) for 10 mins. Supernatant was collected as protein and stored in -80℃ for next step. The protein concentration was ascertained by a commercial BCA kit (MultiSciences, Hangzhou, China). Electrophoresis on SDS-PAGE gels (CW2384, cwbio, China) was used to separate the protein extracts that were transferred onto polyvinylidene difluoride membrane (0.22 μm, Millipore, Merck). The membrane was incubated overnight with the following primary antibodies: anti-claudin-5 (ab15106, 1:1000, Abcam), anti-β- actin (1:1000, proteintech, Wuhan, China) and anti-GAPDH (1:1000, Abcam proteintech, Wuhan, Chian). The immunoreactive bands were visualized using secondary antibodies: goat-anti-mouse (1:8000, proteintech, Wuhan, China) and goat- anti-rabbit (1:8000, proteintech, Wuhan, China), and ECL Chemiluminescence HRP substrate (WBKLS0500, Millipore, Merck) followed by autoradiography. The intensity of the blots was analyzed with ImageJ.

Immunofluorescence and quantification

Brains were post-fixed overnight in 4% PFA before being dehydrated with sucrose solution (10%, 20%, 30%) for 3 days at 4℃. Brains were cut into 50 μm thick sections in the coronal plane. Sections containing DLS and DMS were rinsed with 0.5% Triton X-100 (Solarbio, Beijing) for 5 minutes, and blocked with 5% BSA (Sangon Biotech, Shanghai) for 1 h at room temperature. Then, the sections were incubated with mouse anti-CD31 (ab64543, 1:200, Abcam), chicken anti-GFAP (ab4674, 1:2000, Abcam), rabbit polyclonal anti-Iba1 (ab153696, 1:500, Abcam), rabbit anti-IL17A (A0688, 1:50, Abclonal), and mouse monoclonal anti-CD4 (67786-1-lg,Proteintech)for 48 h at 4℃ in dark. After that, sections were respectively incubated with Alexa-568 donkey anti rabbit IgG (ab175470, 1:1000, Abcam), Alexa-488 donkey anti mouse IgG (ab150105, 1:1000, Abcam), and Alexa-594 goat anti chicken IgG (ab150172, 1:1000, Abcam) for 2h at room temperature. Finally, the sections were mounted on slide with Fluoroshield™ with DAPI (F6057, sigma). Images were captured with a Nikon A1R HD25 confocal microscope system (Nikon, Japan).

Quantitative analysis was performed with the Fiji software. Mean fluorescence intensity (MFI) was applied to claudin-5 fluorescence analysis. The minimum threshold (0-255) was adjusted for each image to exclude background fluorescence (average minimum across all images was 32 ±5).The Analyze-Measure plugin was then applied to all threshold images to collect data and then output the mean gray value (that is integrated density/area). For astrocyte and microglia morphology analysis ,the resulting image was converted to a binary and then skeletonized. The Analyze Skeleton (2D/3D) plugin was then applied to all skeletonized images to collect data and then output the number of endpoints per cell and summarized branch length (μm). The Cell Counter plugin was applied to all images to collect data and then out put the number of CD4^+^IL17A^+^ cells. The final result displayed is the number of positive cells per mm^2^.

Statistical Methods

All data were analysed with GraphPad Prism version 7.0 (GraphPad, San Diego, CA, USA). Data are expressed as the means ± SEM. Measures were analyzed using t-test or one-way analysis of variance (ANOVA) where statistically appropriate. ANOVA was followed with LSD post hoc multiple comparisons. A p value < 0.05 was defined as statistically significant.

**Additional figures and figure legends**

**
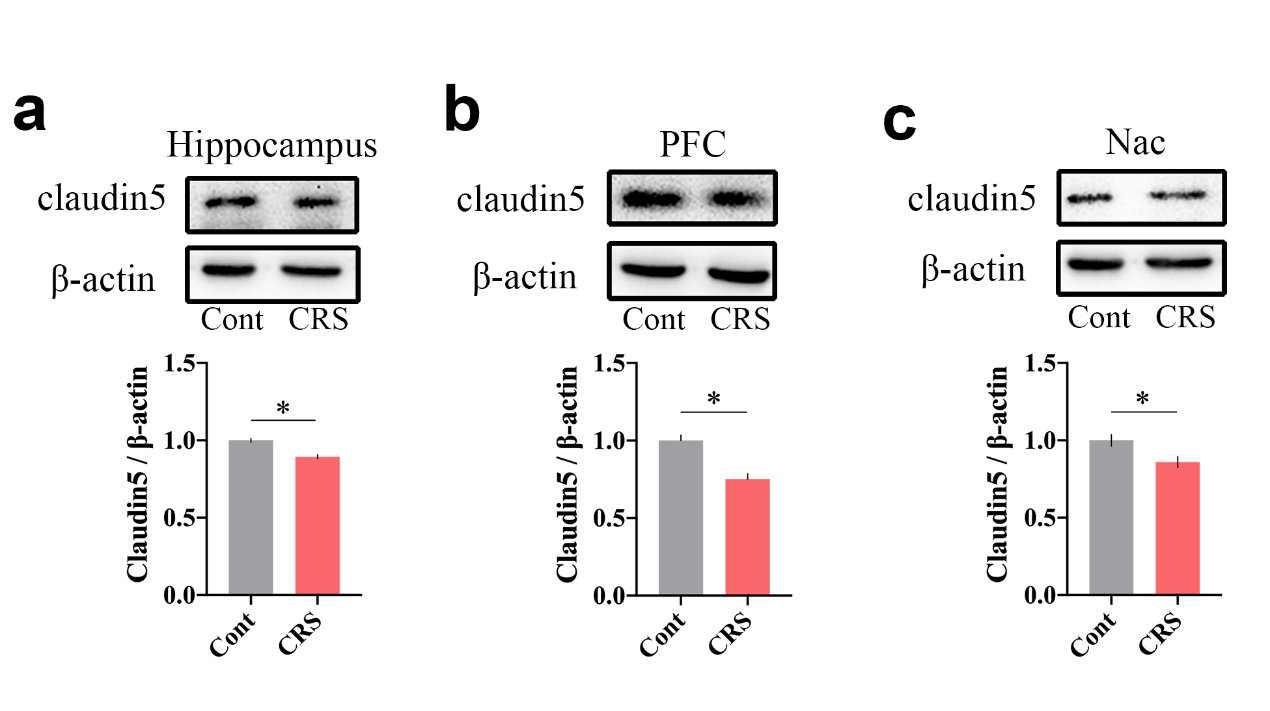
**

**Fig. S1 CRS leads to a** **decrease in claudin-5 expression** in the (a) hippocampus; (b) prefrontal cortex (PFC) and (c) nucleus accumbens (Nac). Data are expressed as mean ± SD, Student’s t-test, **p* < 0.05 compared with control.


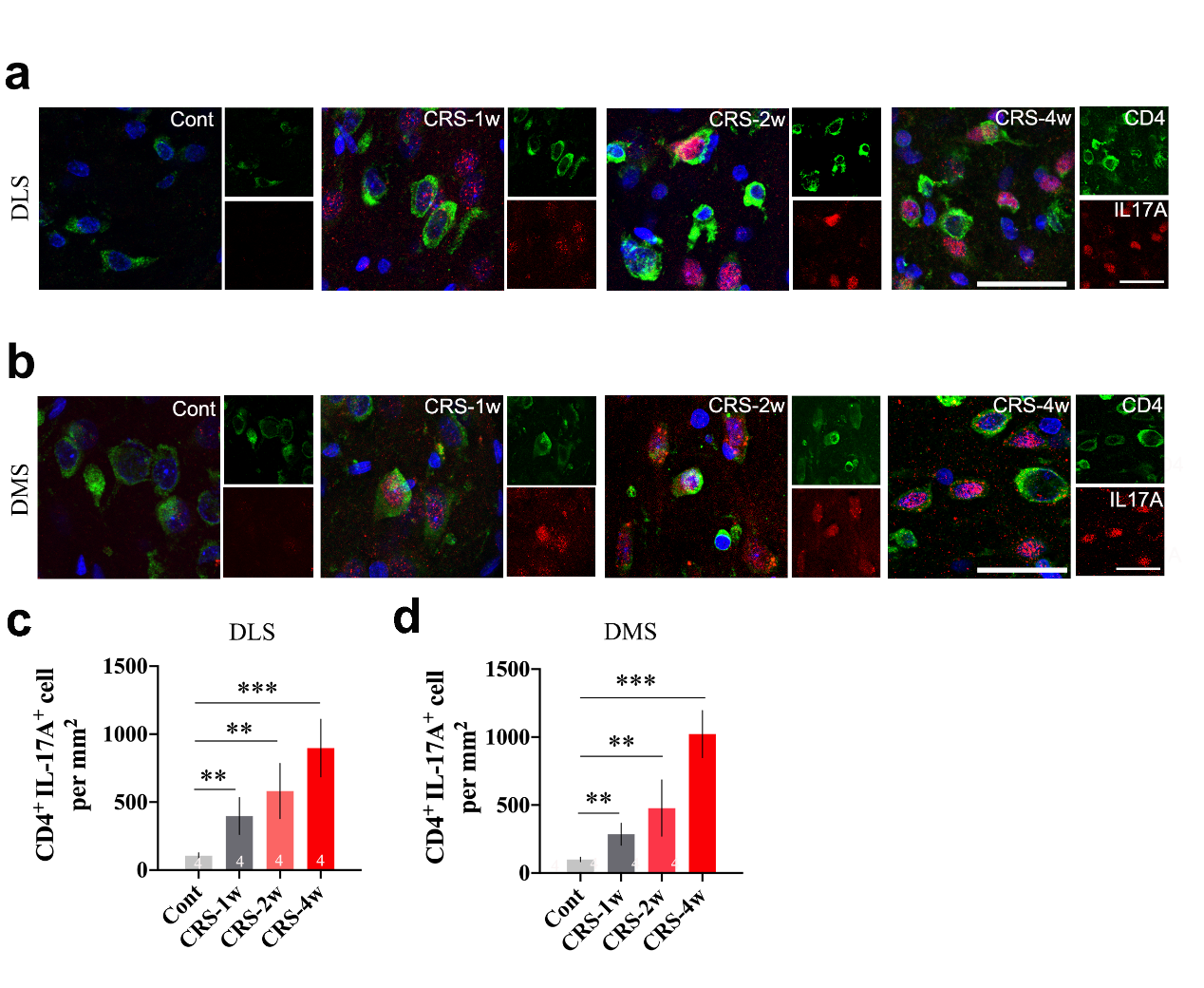


**Fig. S2 CRS increases the accumulation of CD4^+^IL17A^+^ T cells in the dorsal striatum.** Representative confocal images of CD4^+^IL17A^+^ T cells in the (a) DLS and (b) DMS; Cell counting of CD4^+^IL17A^+^ cells in the (c) DLS and (d) DMS. Scale bar, 20 μm. Data are expressed as mean ± SD, Student’s t-test, ***p* < 0.01 and ****p* < 0.0001 compared with control.

**
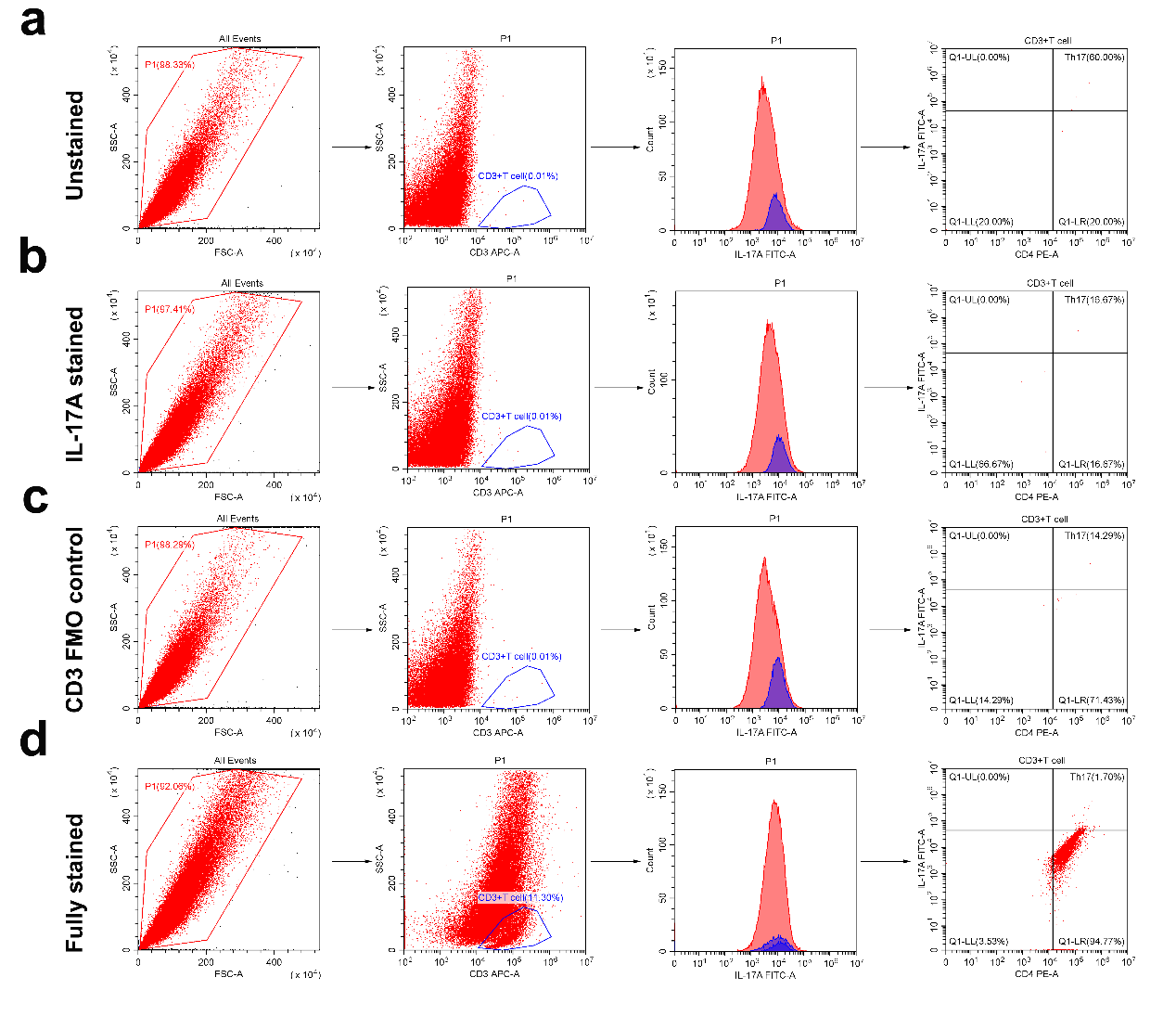
**

**Fig. S3 The gating strategy to** **identify Th17 cells.** CD4^+^IL17A^+^ cells were selected from CD3^+^ T cells and gating with IL-17A staining and FMO control.

**
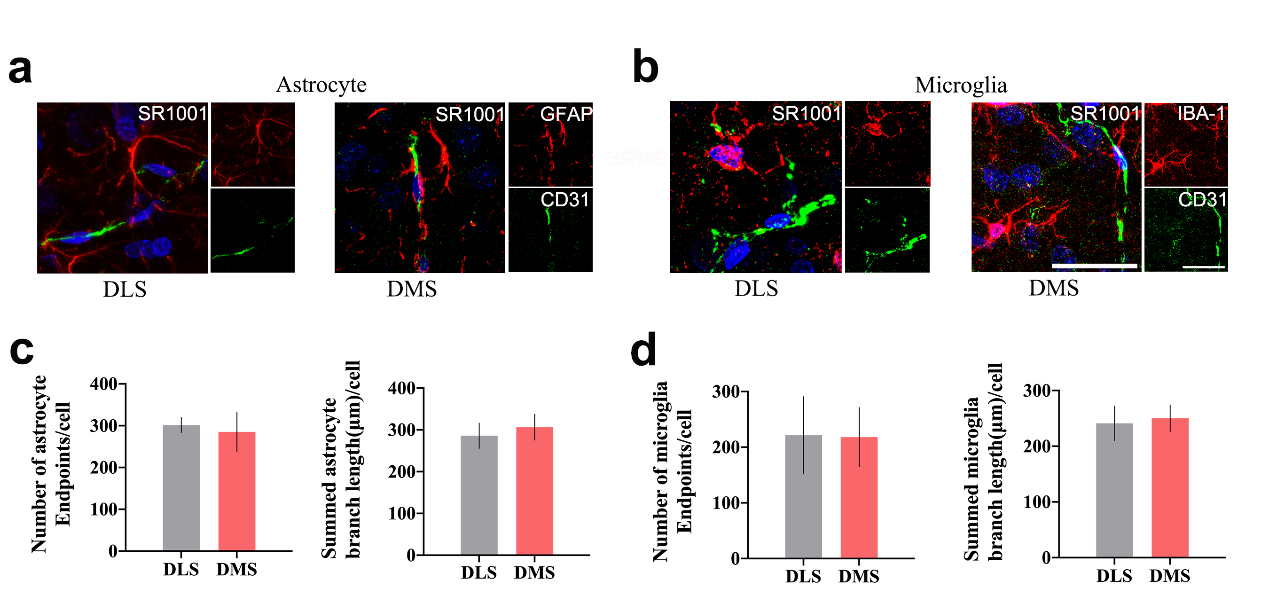
**

**Fig. S4 SR1001 prevents CRS-induced morphological changes in glial cells.** SR1001 prevents CRS-induced morphological changes in (a) astrocytes and (b) microglia near the BBB in the DLS and DMS; (c) and (d) Quantification of morphological changes in astrocytes and microglia. Scale bar, 20 μm. Data are expressed as mean ± SD, Student’s t-test.
